# Epidemiology of carbapenem-resistant *Klebsiella pneumoniae* bloodstream infections after renal transplantation from donation after cardiac death in a Chinese hospital: a case series analysis

**DOI:** 10.1186/s13756-018-0355-8

**Published:** 2018-05-21

**Authors:** Yuxi Wang, Hong Lei, Yuxiang Zhang, Qiwen Yang, Yu Wang, Jiaxing Wang, Cheng Xu, Jinggang Yu, Lili Zhou, Xiaoni Kang, Lei Cui

**Affiliations:** 1 Department of Intensive Care Unit, 309th hospital of Chinese People of Liberation of Army (PLA), No. 17 Heishanhu Road, Haidian District, Beijing, 100091 China; 2 Department of clinical laboratory, Peking Union Medical College Hospital, No.1 Shuaifuyuan Wangfujing, Dongcheng District, Beijing, 100730 China; 3 Department of clinical laboratory, 309th hospital of Chinese People of Liberation of Army (PLA), No. 17 Heishanhu Road, Haidian District, Beijing, 100091 China

**Keywords:** Bacteremia, Infection control, *Klebsiella pneumoniae*, Kidney transplantation, Donation after cardiac death

## Abstract

**Background:**

Although the high mortality rates have been extensively reported worldwide, few studies have investigated the epidemiology of CRKP-BSIs in the early stage after kidney transplantation (KTx) from donation after cardiac death (DCD). We sought to describe the epidemiological and clinical characteristics of cases of carbapenem resistant *Klebsiella pneumoniae* bloodstream infections (CRKP-BSIs) in kidney transplantation recipients (KTRs) from DCD in our hospital.

**Methods:**

A retrospective analysis of clinical data of CRKP-BSIs in KTRs admitted to a Chinese hospital in Beijing, China, between January 1, 2012 and December 31, 2016 was performed. The annual percentage of patients with CRKP, the annual number of total KTRs and KTRs from DCD were determined. The genetic relatedness of the strains was determined by polymerase chain reaction and pulse field gel electrophoresis (PFGE).

**Results:**

During the study period, there were total 947 KTRs in our hospital, including 275 KTRs from DCD. Five incidences of CRKP-BSIs in KTRs were identified, and two of them (Case 1,3) from the same foreign hospital. The incidence of CRKP-BSIs in the early stage (within 3 months) following kidney transplantation (KTx) from DCD was about 1.1% (3/275). In Case 1–3 and 5, the rupture of renal transplant artery was presented on the 40th, 16th, 43th and 74th day after KTx, and in Case 4, the thrombus of renal transplant artery was presented on the 13th day after KTx. Three cases (Case 1,2,5) occurring pneumothorax on the 45th, 51th and 32th day after KTx. Four cases (Case 1–4) received the excision of the transplanted kidney for the treatment. Polymerase chain reaction showed the bands for case 2 were distinctive from other cases. Pulse field gel electrophoresis showed mainly three clusters of the bands for all the isolates.

**Conclusions:**

During the study period, we observed an increase in the occurrence of CRKP-BSIs among KTRs from DCD in our hospital. We demonstrated that rupture/thrombus of the renal transplant artery was associated with CRKP-BSI in the early stage after KTx from DCD. Albeit the low incidence of CRKP-BSI (1.1%) after KTx from DCD, the high mortality (4/5) had been observed from the prognosis of the patients. Thorough surveillance of DCD donors, early identification of CRKP-BSI, necessary preventative measurements and use of appropriate treatments should be the strategy for CRKP-BSI in the early stage after KTx from DCD.

**Electronic supplementary material:**

The online version of this article (10.1186/s13756-018-0355-8) contains supplementary material, which is available to authorized users.

## Background

Renal transplantation has long been deemed to be the best therapeutic option for the treatment of the end stage of kidney failure. However, the deficiency of donors has been the enormous obstacle for the development of renal transplantation in China [1]. The main channel of renal transplantation basically comes from two resources, corpses and living organs [2]. In recent years, under more favorable conditions from aspects of ethics, law and the resources of transplantation, the renal transplantation from donation after cardiac death (DCD) has been widely performed in China. Along with the increasing demanding of organ donors and the technological advancements in the field of organ transplantation, the renal transplantation from DCD has achieved great progression in China, however many unusual complications following transplantation have been reported to date. The rupture or thrombus of the renal transplant artery, and the infection of the surrounding tissues caused by bacteria, especially Gram-negative bacteria [3], or fungus have been regard as two of the hard-handled complications in the early stage (within 3 months) following renal transplantation. Donor-transmitted infection (DTI), including bacterial or fungal infections, has also been frequently reported from kidney recipients from DCD, characterized by Gram-negative predominant donor-derived infection, with higher crude mortality and graft loss than those without donor-derived infection [4].

As the frequently reported pathogen among the donor-transmitted infections associated with kidney transplantation from DCD, Carbapenem resistant Klebsiella pneumonia (CRKP) is a Gram-negative bacterial pathogen responsible for severe diseases, such as septicemia, pneumoniae, urinary tract infection and soft tissue infection, associated with community and hospital acquired infection [5], and characterized by high mortality, high transmissibility and multidrug resistance [6]. It has been reported that high mortality rates, ranging from 20 to 40%, were related to patients with Klebsiella pneumonia bloodstream infection (KP-BSI) [7], but this mortality was reported to rise up to 67.6% for intensive care unit (ICU) patients [8]. Through various mechanisms, such as production of carbapenem-hydrolyzing enzymes (carbapenemases) or changes in membrane permeability, carbapenem resistance can occur [9].

The patients who has undergone the renal transplantation are susceptible to CRKP, attributed to multifaceted factors, ranging from the surgery, the use of immunosuppressant therapy, mechanical ventilation, prolonged use of invasive devices, use of antimicrobial agents and the high sequential organ failure assessment (SOFA) score at admission [10–17]. In the early stage of post-transplantation, especially the first 3 months after renal transplantation, the incidence and severity of illness is directly related to the utilization of immunosuppression and its modulation that primarily intend to lessen the risk of rejection after transplantation [15].

Although the high mortality rates have been extensively reported worldwide, to date, few studies have investigated the epidemiology of CRKP-BSIs in the early stage after renal transplantation from DCD. The study was carried out aiming at describing the epidemiological and clinical characteristics of carbapenem resistant *Klebsiella pneumoniae* bloodstream infections after renal transplantation from donation after cardiac death in our hospital.

## Method

### Study design and population

This was a retrospective cohort study aiming at describing the epidemiological and clinical characteristics of CRKP-BSI after KTx from DCD in the 309th hospital of People Liberation of Army from January 2012 to December 2016. The 309th hospital of People Liberation of Army is a 1430-bed tertiary care teaching hospital with a 20-bed comprehensive Intensive-care-unit (ICU) and a 10-bed surgical Intensive-care-unit (SICU) and approximately 40,000 hospital admissions per year in Beijing, China. Clinical and microbiological characteristic was retrieved from medical records. Case was defined as kidney transplantation (KTx) recipient who developed CRKP-BSIs in the early stage (within 3 months) after KTx, identified by cultures of the patient’s blood specimens and later confirmed by polymerase chain reaction (PCR) during the study period. Patients were followed from KTx to death or automatically discharge or discharge upon recovery.

In the analysis of the profile of utilization of immunosuppression drugs following renal transplantation, we evaluated age; gender; cold ischemia time (CIT); immunosuppression drugs (calcineurin inhibitor, adjuvant drug and monoclonal and polyclonal antibodies) and etiology of chronic renal failure (CRF), including systemic arterial hypertension (SAH), diabetes, glomerulonephritis (GMN), IgA nephropathyand charlson comorbidity index (CCI) score.

In the analysis of clinical characteristics of the CRKP-BSIs, we analyzed age; gender; ureteral stent; indwelling catheters; use of continuous renal replacement therapy (CRRT); blood transfusion; infection related to invasive devices; intervention for focus control (< 48 h); polymicrobial infection; acute rejection; cytomegalovirus disease; carbapenem resistant klebsiella pneumonia Bloodstream infection (CRKP-BSI); complication of infection; time (days) from kidney transplantation to the excision of the transplanted kidney; time (days) from kidney transplantation to ICU department; time (days) from kidney transplant to rupture/thrombus of the artery of the transplant kidney; time (days) from kidney transplant to the first bloodstream infection (BSI); time (days) from kidney transplantation to death/automatically discharge/discharge upon recovery; Length of stay (LOS, days) after first BSI onset; ICU-LOS (days); total LOS (days); Pitt bacteria score (PBS) on the onset of first BSI; Sequential Organ Failure Assessment (SOFA) score on the onset of first BSI; serum creatinine (μmol/L) before renal transplant; serum creatinine (μmol/L) after renal transplant; serum creatinine (μmol/L) at first BSI; serum creatinine (μmol/L) at the 7th and 14th day after first BSI; combinational antibiotic therapy; the total medical costs (¥) and prognosis.

The research project has been approved by the Ethics Committee and has therefore been performed in accordance with the ethical standards laid down in the 1964 Declaration of Helsinki and its later amendments.

### Microbiology

With an automated susceptibility testing system (VITEK; bioMérieux, Marcy l’Étoile, France), carbapenem-resistant strains of *K. pneumoniae* were identified. Minimum inhibitory concentration (MICs) were interpreted according to the Clinical and Laboratory Standards Institute breakpoints [16]. Minocycline/Tigecycline susceptibility was interpreted according to breakpoints approved by the US Food and Drug Administration [17]. All of the isolates were submitted to enterobacterial repetitive intergenic consensus polymerase chain reaction (ERIC-PCR) for the genetic relatedness analysis. ERIC-PCR specific primers (ERIC-1R, 5’-ATGTAAGCTCCTGGGGATTCAC-3′; ERIC-2, 5’-AAGTAAGTGACTGGGGTGAGCG-3′) were based on a report by Versalovic et al. [18]. An amplification reaction was performed in a volume of 50 μl containing 5 μl of 10 × PCR buffer, 10 pmol of each ERIC primer, 1.5 mM MgCl_2_, 0.2 mM of each deoxynucleoside triphosphate (dATP, dGTP, dCTP, and dTTP), 3 μl dimethylsulfoxide, 3 μl of chromosomal DNA solution, and 1 unit of Taq polymerase (Invitrogen; Thermo Fisher Scientific Corporation, Carlsbad, Canada). The DNA concentration range was about 20 to 50 ng of genomic DNA per microliter. Cycling conditions were as follows: denaturation at 95 °C for 3 min, followed by amplification for 35 cycles of 95 °C for 0.5 min, 51.2 °C for 1 min and 72 °C for 2 min, followed by a final extension step at 72 °C for 5 min. The PCRs were performed in a BIO-RAD T100 thermal cycler. Electrophoresis of the PCR products were carried out in 1% agarose gel. All gels were stained with ethidium bromide (1 μg/ml). Amplified DNA fragments of specific sizes were visualized with an ultraviolet (UV) transilluminator and photographed.

Furthermore, the genetic relatedness of the isolates obtained from 5 CRKP-BSI strains were determined by the pulse field gel electrophoresis (PFGE), and the bands showed on the PFGE underwent the clustering analysis via the BioNumeric software version 6.0 (Applied Maths NV, Sint-Martens-Latem, Belgium).

### Definition

The clinical and microbiological outcomes were analyzed. BSI was defined according to the Centers for Disease Control and Prevention guidelines (available at: http://www.cdc.gov/nhsn/pdfs/pscmanual/17pscnosinfdef_current.pdf). BSI onset was defined as the collection date of isolate. The probable infectious source was determined on the basis of the microbiological results and the analysis by at least 2 physicians. Crude mortality was defined as death occurring after the collection of the first blood culture positive for *K. pneumoniae*. Mortality attributable to BSI was defined by clinical evidence of active infection and positive cultures, or when death occurred as the result of organ failure that developed or deteriorated during the onset of infection [19]. Appropriate treatment was defined as ≥48 h of treatment with at least one antimicrobial agent that has been proven in vitro activity against CR-KP strains [20]. Septic shock was defined as sepsis associated with organ dysfunction and persistent hypotension despite volume replacement [21].

## Results

### Increased percentage of CRKP, especially among ICU patients

**Fig. 1:**
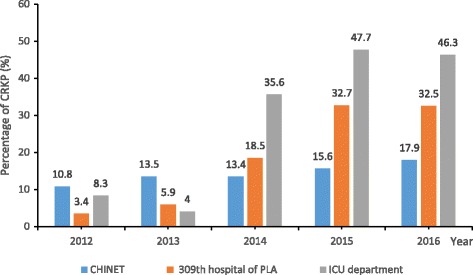
CRKP among identified KPNs from January 2012 to December 2016. Blue bar, percentage of CRKP from CHINET; Orange bar, percentage of CRKP in 309th hospital of PLA; Grey bar, percentage of CRKP in the ICU department of 309th hospital of PLA

*Klebsiella pneumoniae* (KPN) strains were collected and analyzed from January 1, 2012, to December 31, 2016 in our hospital. Among these strains, carbapenem resistant *Klebsiella pneumoniae* (CRKP) strains were identified via various cultures, coming to the result of the percentage of CRKP isolates. During this 5-year period, 853 episodes of *Klebsiella pneumoniae* (KPN) in our hospital were obtained, and 18.8% (160/853) of these strains were CRKP isolates, while 36.8% (74/201) of the K pneumoniae strains identified from ICU were CRKP isolates. The annual rate of CRKP in clinical isolates in China, obtained from the CHINET surveillance of bacterial resistance [22, 23], has shown the steadily increased trend of the percentage of CRKP during 2012 to 2016 nationally. As Fig. 1 shown, the percentage of CRKP isolates in our hospital significantly increased from 3.4% in 2012 to 32.5% in 2016, with the highest rate obtained in 2015, up to 32.7%. Compared with the trend of the percentage of CRKP isolates in our hospital, the percentage of CRKP in ICU department initially declined from 8.3% in 2012 to 4% in 2013, then significantly increased from 4% in 2013 to 46.3% in 2016, with the highest rate reached 47.7% in 2015. In contrast to the above trends, the rate of CRKP rising from 10.8% in 2012 to 17.9% in 2016 was observed in data obtained from the CHINET surveillance program.

Owing to the deficiency of donors, the total number of kidney transplants was declined from 208 in 2012 to149 in 2014 in our hospital. With the widely application of kidney transplantation from DCD, it’s rising from 149 in 2014 to 232 in 2016. As for the kidney transplantations from DCD, although kidney transplants from DCD was in absence in the first 2 years of the study, however, it was rising from 18 in 2014 to 170 in 2016. During the study period, there were only 5 CRKP-BSIs after renal transplantation from DCD, including 1 case (Case 1) in 2015 and 4 cases (Case 2–5) in 2016. Noticeably, both of the renal transplantations for Case 1 and Case 3 performed in the same foreign hospital. Therefore, apart from these renal transplantations that performed in foreign hospital, the incidence of CRKP-BSI after renal transplantation from DCD was about 1.1% (3/275) from January 1, 2012 to December 31, 2016 in our hospital.

### The application of immunosuppression regimen during the perioperative period

The application of immunosuppressive regimen basically complied with a routine regimen, however varied case by case. According to the routine regimen, Mycophenolate Mofetil (MMF) was orally administered (1 g, tid) preoperatively, combined with the intravenous infusion of Basiliximab (20 mg), followed by the second administration of Basiliximab on the 4th day postoperatively, or the RATG (rabbit anti-thymic globulin) (1.25–2.5 mg/kg, qd) for 5 days. During the preoperative period, the glucocorticoid (1 g) was intravenously given, and glucocorticoid (0.5 g) was continually given for 3 consecutive days postoperatively. Tacrolimus, mycophenolate mofetil and glucocorticoid were taken orally for a long time for induction -or anti-rejection therapy. Based on the cases, the most frequently used immunosuppressive drugs were Tacrolimus, MMF and Basiliximab owing to the superiority of the therapeutic efficacy and relative drug safety [24–26]. Undoutedly, the application of immunosuppressive drugs had altered the immunological status of the 5 patients, causing the subsequent occurence of CRKP-BSI after KTx.

### The clinical characteristics of the 5 CRKP-BSIs

The mean age of these 5 patients was 48.6 years (median: 47, range: 44–54 years), and 3/5 of the patients were male. The etiology of chronic renal failure (CRF) for the 5 CRKP-BSIs included systematic arterial hypertension (SAH), diabetes, glomerulonephritis (GMN) and IgA nephropathy, with the most common being SAH (see Additional file 1: Table S1). With respect to case 1 and 2, SAH was recognized as the etiology of CRF, and SAH, GMN and IgA nephropathy were viewed as the etiology of CRF for case 3 and 4. In contrast to the above cases, SAH and Diabetes turned out to be the etiology of CRF for case 5.

Of the 4 cases (Case 1–3,5), the rupture of renal transplant artery was presented on the 40th, 16th, 43th and 74th day after KTx respectively. The thrombus of renal transplant artery was presented on the 43th and 13th day after KTx for Case 3 and Case 4. Additionally, on the 45th, 51th and 32th day after KTx, three cases (Case 1,2,5) occurring pneumothorax. Four cases (Case 1–4) received the excision of the transplanted kidney for the treatment. Detailed description of the clinical characteristics of the 5 CRKP-BSIs is shown (see Additional file 2: Table S2 and Additional file 3: Table S4).

### Antibiotic susceptibility of 5 CRKP-BSIs following renal transplantation from DCD at the first bloodstream infection

In the whole picture, the antibiotic susceptibility of Case 2 distinguished from other cases in line with the genetic distinction (see Additional file 4: Table S3). With regards to Case 2, The MIC for Imipenem and Amikacin was 4_R and≦2_R, and it did not higher than 4_R for other antibiotics, except for Gentamicin (≧16_R). All the other cases showed similar resistance to antibiotics (MIC no lower than 2_R), except for Minocycline/Tigecycline. Based on the disc diffusion method, the result of antibiotic susceptibility to Minocycline/Tigecycline (Case1,3,5) showed 13_S, 18_S and 24_S, thereby suggesting the possibility of the utilization of the Tigecycline that has similar structure as Minocycline.

### ERIC-PCR analysis of CRKP ICU isolates

**Fig. 2:**
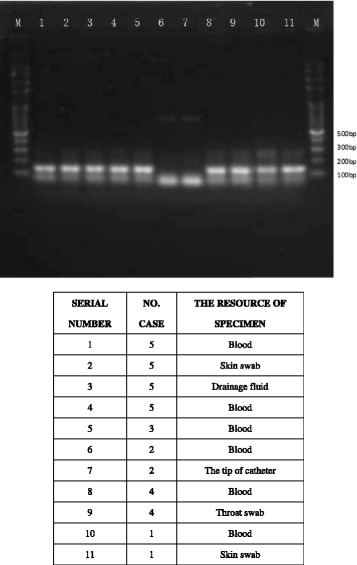
The ERIC-PCR gene confirmation of various cultures from case 1 to 5

To further determine the genetic relatedness of CRKP-BSI strains in ICU, we selected 11 CRKP ICU isolates for enterobacterial repetitive intergenic consensus (ERIC) PCR based genetic analysis. The obtained results indicated that these isolates belonged to two different clonal types (Fig. 2). The predominant clonal type was shown to be A type (*n* = 9) with the size about 150 kb. The other clonal type was B type (*n* = 2) with the size about 50 kb, belonging to the isolate from Case 2, indicating that the *Klebsiella pneumoniae* strain from Case 2 was different from the other strains. The result suggested that clone spread may contribute to the nosocomial dissemination of CRKP.

### PFGE analysis of CRKP ICU isolates

**Fig. 3:**
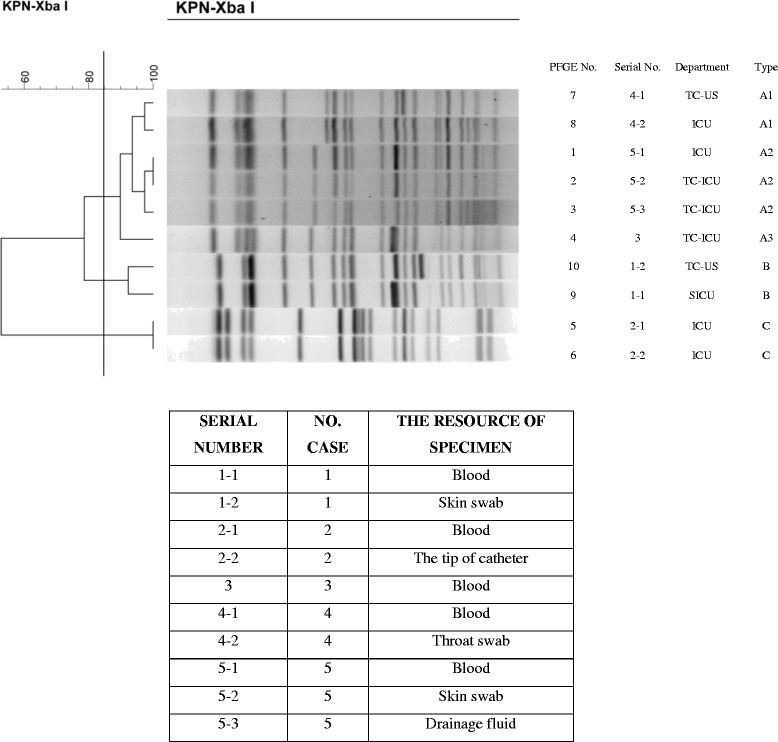
PFGE analysis of various CRKP-BSI isolates. Genomic DNA was digested using Xba I enzyme and subjected to PFGE. The bands were further analyzed by BioNumeric software version 6.0. Abbreviation: *ICU* Intensive care unit *SICU* Surgery Intensive Care Unit, *TC* Transplantation Center, *TC-ICU* Transplantation Center Intensive Care Unit, *TC-US* Transplantation Center Urinary Surgery

To further determine the genetic relatedness of CRKP-BSI strains in ICU, we randomly selected 10 CRKP-BSI ICU isolates for PFGE analysis. Through the clustering analysis, the obtained results indicated that these isolates belong to three different clonal types, based on the 85% similarity (Fig. 3). The predominant clonal type was shown to be A type (6 isolates [60%], including A1-subtype [*n* = 4], A2-subtype [*n* = 1] and A3-subtype [n = 1]), coming from the Transplantation Center Urinary Surgery, Intensive Care Unit and Transplantation Center Intensive Care Unit, respectively. The B type (2 isolates [20%]) was obtained from Transplantation Center Urinary Surgery and Surgery Intensive Care Unit. The C type (2 isolates [20%]) was collected from Intensive Care Unit.

## Discussion

*Klebsiella pneumoniae* (KP), with the characteristics of hypervirulent, highly transmissible and high mortality, has attracted a lot of attention in recent years, together with the worldwide spread of multidrug resistant KP strains. As the most lethal type of infection, *Klebsiella pneumoniae* bloodstream infection (KP-BSI), with the alarming high mortality in ICU, has been placed in the focus of various researches worldwide, showing the complex relationships with various factors, ranging from solid organ or stem cell transplantation, the frequent use of invasive devices, surgery, hematological malignancy to the previous use of antibiotics. In China, the CHINET data showed that the rate of carbapenem resistance of *K. pneumoniae* isolates rose steadily from 10.8% in 2012 to 17.9% in 2016. The rate of CRKP has also increased dramatically worldwide over the past 10 years [27], and in the areas of high CRKP endemicity, such as United States, Israel, Italy, and Greece, the percentage of CRKP-BSI was shown to be 18–68% [19, 28–33]. Compared with these results, we observed a rapid increase in CRKP-BSI rates in our hospital after 2013, and the obtained data showed that these rates were higher than the average rates in China after 2014, especially among ICU patients, rising up to 47.7% in 2015, but declined to 46.3% in 2016 owing to the preventative measurements for the infection. In this research, the incidence of CRKP-BSI in the early stage (within 3 months) after renal transplantation from DCD was about 1.1% (3/275) in our hospital, approximating to the result of nationwide surveillance study of the incidence of carbapenem-resistant gram negatives (CRGN) in Italian transplant recipients performed by Lanini S et al., which comes to the Incidence rates were 1.23, 0.51 and 0.16 per 1000 recipient-days at 0–30, 31–60, and 61–90 days after solid organ transplantation (SOT), respectively [31].

**Fig. 4:**
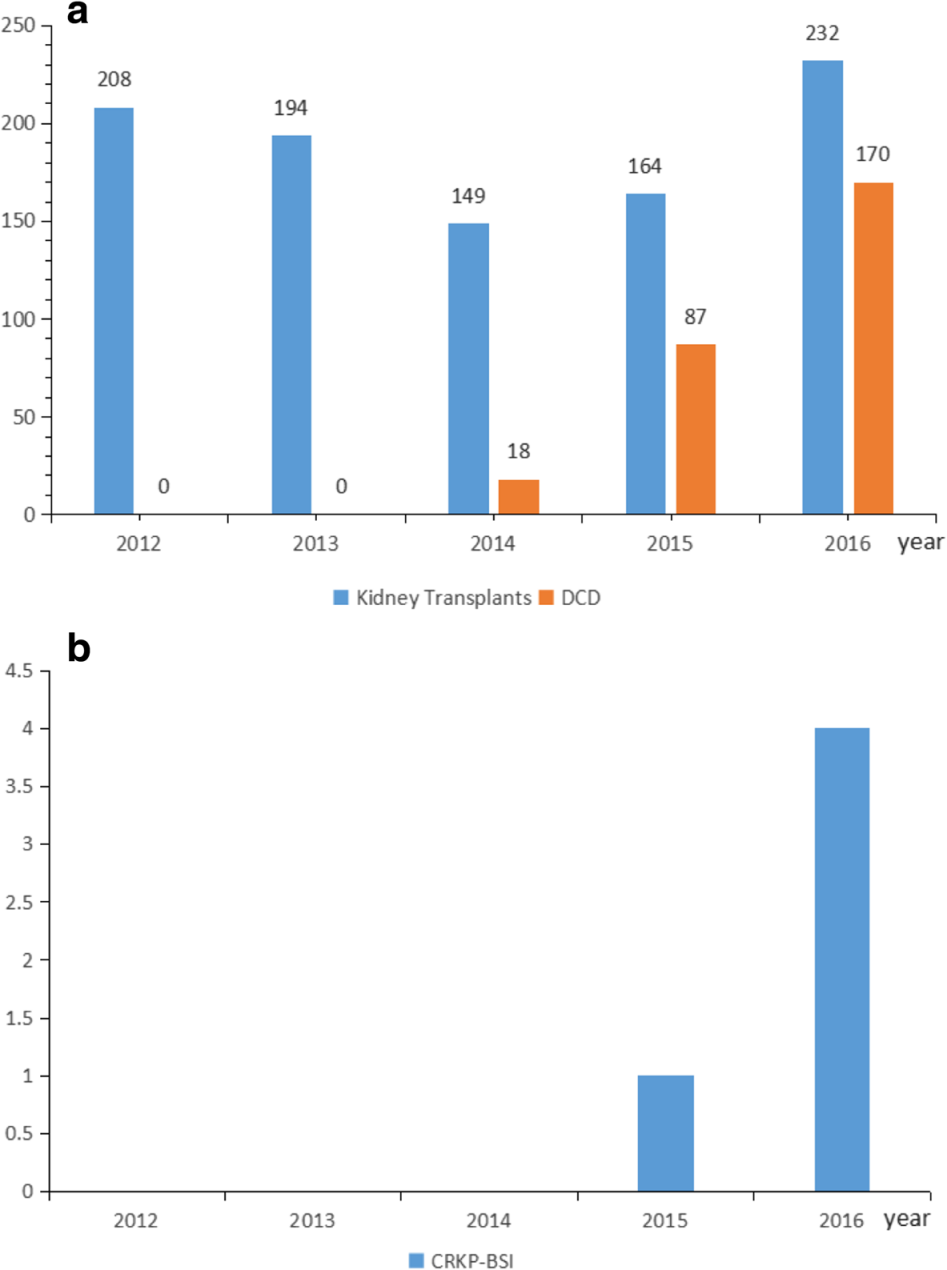
**a** The total number of kidney transplants and the number of donation after cardiac death (DCD) in the hospital from 2012 to 2016. **b** The number of isolates of CRKP-BSI after the kidney transplants in the hospital from 2012 to 2016

Just as Fig. 4 shown, considering the renal transplantation performed in the foreign hospital and the confirmed infection of CRKP following renal transplantation, there is a possibility that Case 1 plays the role as the exogenous source, associated with the following series of CRKP-BSIs in our hospital in the next year. The result of ERIC-PCR has substantiated the supposition that there is certain level of nosocomial clonal spread, disseminating the pathogen from the exogenous resources, and highlighting the necessity of implementation of thorough surveillance of recipients of renal transplantation before transferring from foreign hospital.

**Fig. 5:**
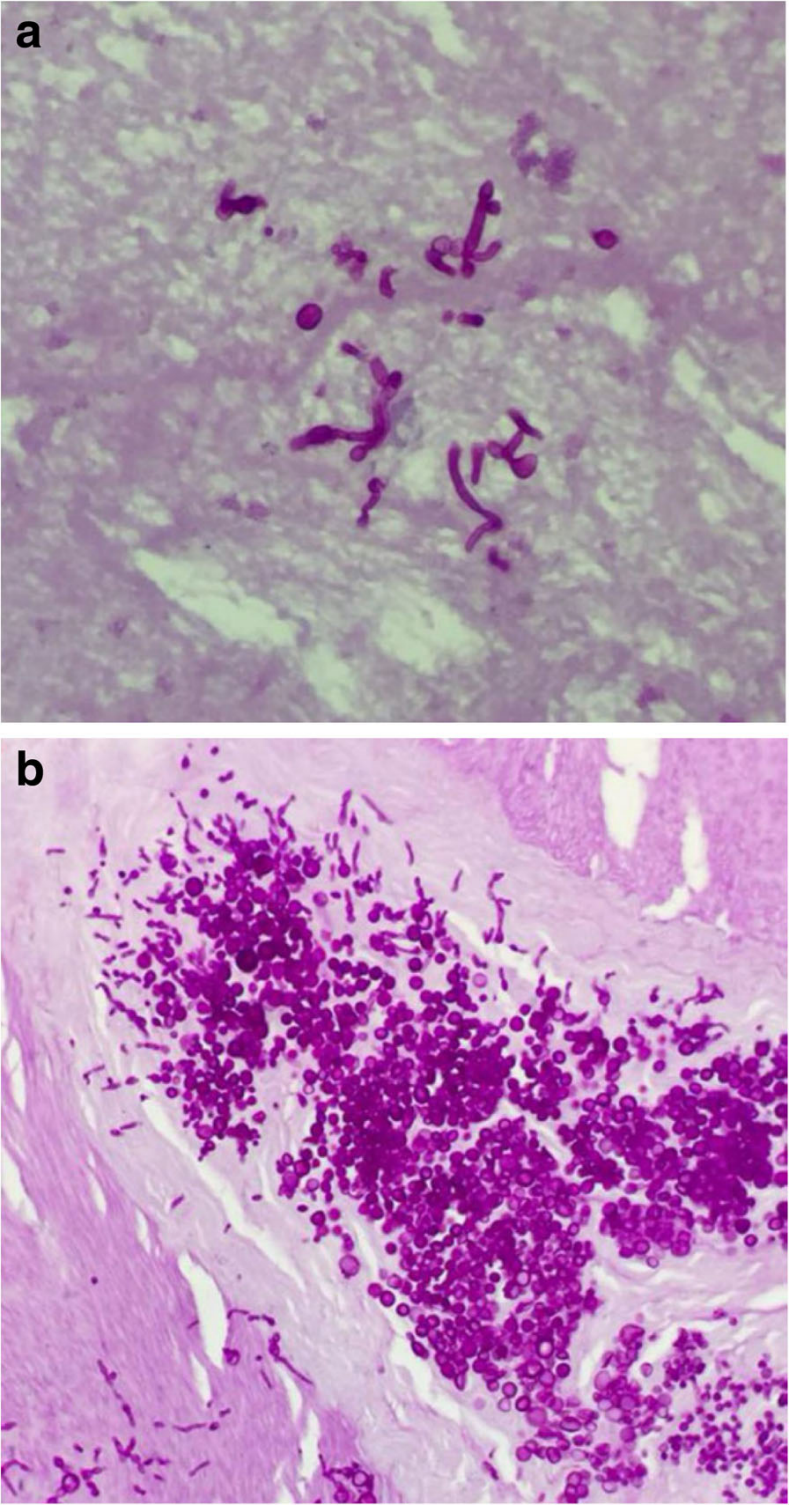
The pathological confirmation of aspergillus invasion from the specimen extracted during the surgery from Case 2. **a** (Within the anastomosis of external iliac artery and the renal transplant artery) Multiple fungi contained in the specimen, with the form partially like Aspergillus and partially like Cryptococcus. **b** (Tissue resection specimens from the transplanted kidney and renal hilus) The chronic inflammation overlapping with acute inflammation and exudation were shown around the renal artery. A small amount of fungal mycelium and spores was shown in the exudate. Compared with the previous biopsy, morphology was consistent with Aspergillus. Acute inflammatory cell infiltration in the renal artery wall was observed, showing myxoid degeneration and significant proliferation of smooth muscle cells. A small amount of lymphocyte infiltration was presented within the renal interstitium. There is no significant degeneration within renal interstitium. Urinary tract epithelial mucosal chronic inflammation was observed in ureter and renal pelvic

Clinical characteristics associated with CRKP-BSI patients after renal transplantation in our study has been attributed to the various aspects. All of the patients had received the renal transplant from donation after cardiac death (DCD). Undoubtedly, compared with the living donor, as the supplement mean for transplantation, the renal transplants from DCD has satisfied the increasing demanding of the resource of kidney nowadays and accelerated the advancement of technology and conception of renal transplants. However, there is controversy with respect to the infection risk of renal transplantation from DCD worldwide. Sousa SR et al. had come to conclusion that renal transplants from decreased donors and a prolonged cold ischemia time were important risk factors for infections following renal transplantation [32]. Fernández-Ruiz M et al. performed a prospective cohort study of 291 KT patients, drawing the conclusion that uncontrolled DCD policies were safe in terms of the risk of post-transplant infection in spite of higher rate of delayed graft function (DGF) and the use of ATG-containing sequential therapy [33]. In our study, we had observed that in the early stage after renal transplantation (3 months), the CRKP induced rupture/thrombus of the artery of the transplanted kidney occurred. During the course of treatment, the predominant features of all the cases included the placement of catheters, the use of antibiotics, renal transplantation, prolonged stay of ICU department, and the high dose immunosuppressive therapy, predisposing to the vulnerability of CRKP-BSI. Additionally, the most common site of CRKP isolation, in our study, was organ-space surgical site, followed by urinary tract and pneumoniae. In a study of LTx recipients, in the first month after transplantation, 71% of the KPC-Kp-HAIs had abdominal infections [34]. In other studies of KTx recipients, the most common foci of infection have been found to be the surgical wound and the urinary tract [35]. These studies suggest that various site/type of infection, for instance abdominal effusion, surgical wound, urinary tract and so on, can be the susceptible sites of CRKP-BSIs as the manifestation of the systemic infection on specific site, serving as part of the systemic infection. The peri-renal infection can lead to the rupture or thrombus of the renal transplant artery, with the consequence of the excision of transplanted kidney, and be reckoned as the severe complication after kidney transplantation. In our research, all of the five cases have close relationship with CRKP, with one case (Case 2) associated with the mixed infection of CRKP and aspergillus that confirmed by the pathologic report (Fig. 5), therefore administrating different antibiotic regimen with the wide coverage for aspergillus. Xu MJ et al. reported 5 KTRs occurring the infectious rupture of renal transplant artery, with 1 KTR infected with CRKP, while other KTRs infected with fungus [36]. Wan Q et al. reported 3 of 154 KTRs developed confirmed donor-derived CRKP infections, with 1 CRKP-BSI and 1 CRKP died due to the rupture of renal transplant artery, coming to the conclusion that *Klebsiella pneumoniae* was the most probable causative microorganism leading to the episode of renal artery rupture [4].

When it comes to the treatment for CRKP-BSI after renal transplantation, a multidisciplinary therapy, combined with the surgical therapy and the combinational antibiotic therapy, especially regimens including a carbapenem, has been proven beneficial for the patient, demonstrated in several retrospective studies of general population [37, 38]. From the cases, we learn that it is critical to obtain the positive result of culture in the early stage of treatment, guiding the following definitive antibiotic therapy. Usually, a combined treatment (Tigecycline + Carbapenem, Tigecycline + Fosfomycin, Fosfomycin + Aminoglycoside, Third generation cephalosporin +β-lactamases inhibitors etc.) can be prescribed for the treatment of CRKP-BSI. If permitting, high dose Tigecycline (100 mg, q12h after 200 mg loading dose) scheme is worth recommending to bring a satisfactory outcome for the patient [39], and in Case 1 and 5, the respective scheme had been applied. Based on the differential result of antibiotic susceptibility and the evidence of growth of aspergillus on the transplanted kidney, the renal transplant artery and external iliac artery, plus with the infection of Candida, the antibiotic regimen, consisted of Meropenem, Tigecycline, Amikacin, Voriconazole and Amphotericin B, was prescribed for Case 2. Important of all, utilization of properly mini-invasive surgical approaches, for example the indwelling catheter drainage for the abdominal fluids, can alleviate the severity of the disease to a large extent and extend the survival, combined with the appropriate combined antibiotic therapy. Last but not least, the cooperation between multiple medical departments is also vital for the treatment, not only demonstrating the necessary coordination between departments for the therapy, but also enhancing the efficacy of treatment and avoiding the incompleteness and shortage of medical knowledge and techniques for one department.

In United states and Europe, there are myriad of clinical reports regarding the application of Ceftazidime, Avibactam and Polymyxin B, obtaining surprising and satisfactory therapeutic effect. Nevertheless, Polymyxin B came into Chinese market in December 2017, while Avibactam had not appeared on the Chinese market yet. In the absence of international newly developed drugs, the antibiogram for treating CRKP fundamentally was based on the combined treatment, consisted of Carbapenem/Aminoglycoside/Fosfomycin and Tigecycline, until now in China. In the near future, in light of pharmacokinetic/pharmacodynamic (PK/PD) profile of the CRKP-BSIs after KTx, it is worth adopting the multi-drugs combinational antibiotic strategy to elevate the cure rate for the KTRs infected with CRKP-BSI, taking advantage of the beneficial therapeutic approach including the high-dose prolonged-infusion (3–4 h) regimen to drive the PK/PD to acceptable exposures [40].

Our study has several limitations. Clinical data were obtained retrospectively from medical records, and therefore, there may exist some differences in physician practices or accuracy of information. Additionally, the data for patients who may have had significant BSI symptoms such as septic shock and hyperpyrexia, but were not tested owing to the patient refusal or because their blood culture was negative, were not included. Finally, this was a single-center study, showing all the 5 recorded CRKP-BSIs with detailed clinical analysis. A large-scale of study on the clinical characteristics of CRKP-BSIs after renal transplantation from donation after cardiac death is underway to provide more evidence on the clinical significance of the hard-handled multi-resistant superbug. Most important of all, the investigation of transmission of bacterial or fungal infection to kidney transplant recipients from DCD has also been on the way to clarify the etiology of CRKP-BSI after renal transplantation and help designate an effective strategy to eliminate the possibility of bacterial infection before transplantation for the surgeons and physicians in the ICU and transplant department.

As the recipients of renal transplantation from donation after cardiac death, many factors can interact modifying the risk for infection, such as the immunosuppression profile used, the post-operative care, and the profile of epidemiological exposure to the susceptibility to CRKP [32]. It is critical to perform thorough surveillance of the source of kidney before transplantation, the protection of integrity of skin and mucosal barriers in the critically ill patients or the patients undergoing invasive procedures, and all necessary contact precautions should be employed by hospital staff caring these high-risk patients [41]. In conclusion, the thorough surveillance of sources of kidney before transplantation, early clinical identification of the infection of carbapenem resistant klebsiella pneumonia, the necessary preventative measurements and the use of the appropriate treatment are the reasonable strategy to treat the CRKP-BSI in the early stage after renal transplantation from DCD.

## Conclusion

An increase in the occurrence of CRKP-BSIs among KTRs from DCD in our hospital was observed in our hospital during January 1, 2012 to December 31, 2016. Rupture/thrombus of the renal transplant artery was demonstrated to be associated with CRKP-BSI in the early stage after KTx from DCD. Despite the low incidence of CRKP-BSI (1.1%) after KTx from DCD, the high mortality (4/5) had been observed.

## Additional files

Additional file 1: **Table S1.** Demographic characteristics and utilization of immunosuppression drugs of patients following renal transplantation. (XLSX 10 kb)

Additional file 2: **Table S2.** Summary of carbapenem-resistant *Klebsiella pneumoniae* (CR-KP) isolation site, episodes of sepsis, antibiotic treatments adopted, outcome, excision of transplanted kidney and cause of death. (XLSX 9 kb)

Additional file 3: **Table S4.** Analysis of clinical characteristics of 5 kidney recipients infected with *Klebsiella pneumoniae* carbapenem resistant *K. pneumoniae*. (XLSX 12 kb)

Additional file 4: **Table S3.** Antibiotic susceptibility of 5 stains of carbapenem-resistant *K. pneumoniae* isolated from first bloodstream infections in kidney transplant recipients. (XLSX 9 kb)

## Abbreviation

A: Abdominal cavity
AMB: Amphotericin B
AMK: Amikacin
ATP: Adenosine triphosphate
B: Blood
CCI: Charlson comorbidity index
CHINET: China bacterial resistance monitoring network
CIT: Cold ischemia time
CPS: Capsular polysaccharide
CRF: Chronic renal failure
CRGN: carbapenem-resistant gram negatives
CR-KP: carbapenem-resistant *Klebsiella pneumoniae*
CRKP-BSI: Carbapenem-resistant *Klebsiella pneumoniae* bloodstream infection
CRRT: Continuous renal replacement therapy
CTP: Cytidine triphosphate
DCD: Donation after cardiac death
DGF: Delayed graft function
DNA: Deoxyribonucleic acid
DTI: Donor-transmitted infection
E: External iliac artery
ERIC-PCR: Enterobacterial repetitive intergenic consensus polymerase chain reaction
G: Gallbladder
GMN: Glomerulonephritis
GTP: Guanosine triphosphate
I: Incision
ICU: Intensive care unit
IPC: Infection prevention and control policy
KP: *Klebsiella pneumoniae*
KP-BSI: *Klebsiella pneumoniae* bloodstream infection
KPC-Kp-HAIs: *Klebsiella pneumoniae* carbapenemase-producing *Klebsiella pneumoniae*-Hospital acquired infections
KPN: *Klebsiella pneumoniae*
KTR: Kidney transplantation recipient
KTx: Kidney transplantation
L: Lung
LOS: length of stay
LTx: Liver transplantation
MEM: Meropenem
MIC: Minimum inhibitory concentration
MMF: Mycophenolate mofetil
NA: Non-applicable
NIT: Nitrite
O: Organ-space surgical site
OSSS: Organ-space surgical site
PBS: Pitt bacteria score
PCR: Polymerase chain reaction
PLA: Chinese people of liberation of army
SAH: Systemic arterial hypertension
SICU: Surgery intensive care unit
SOFA: Sequential organ failure assessment
SOT: Solid organ transplantation
T: Thoracic cavity
TC: Transplantation center
TC-ICU: Transplantation center intensive care unit
TC-US: Transplantation center urinary surgery
TGC: Tigecycline
Ti: Tip of catheter
TTP: Thymidine triphosphate
U: Urinary tract
UV: Ultraviolet
VOR: Voriconazole

## References

[1] Huang J, Millis JM, Mao Y, Millis MA, Sang X, Zhong S (2012). A pilot programme of organ donation after cardiac death in China. Lancet.

[2] Huang J, Mao Y, Millis JM (2008). Government policy and organ transplantation in China. Lancet.

[3] Yeşilkaya A, Azap ÖK, Demirkaya MH, Ok MA, Arslan H, Akdur A (2013). Bloodstream infections among solid organ transplant recipients: eight years’ experience from a Turkish university hospital. Balkan Med J.

[4] Wan Q, Liu H, Ye S, Ye Q (2017). Confirmed transmission of bacterial or fungal infection to kidney transplant recipients from donated after cardiac death (DCD) donors in China: a single-center analysis. Med Sci Monit.

[5] Podschun R, Ullmann U (1998). Klebsiella spp. as nosocomial pathogens: epidemiology, taxonomy, typing methods, and pathogenicity factors. Clin Microbiol Rev.

[6] Gu D, Dong N, Zheng Z, Lin D, Huang M, Wang L (2018). A fatal outbreak of ST11 carbapenem-resistant hypervirulent Klebsiella pneumoniae in a Chinese hospital: a molecular epidemiological study. Lancet Infect Dis.

[7] Meatherall BL, Gregson D, Ross T, Pitout JD, Laupland KB (2009). Incidence, risk factors, and outcomes of Klebsiella pneumoniae bacteremia. Am J Med.

[8] Delle Rose D, Sordillo P, Gini S, Cerva C, Boros S, Rezza G (2015). Microbiologic characteristics and predictors of mortality in bloodstream infections in intensive care unit patients: a 1-year, large, prospective surveillance study in 5 Italian hospitals. Am J Infect Control.

[9] Nordmann P, Cuzon G, Naas T (2009). The real threat of Klebsiella pneumoniae carbapenemase-producing bacteria. Lancet Infect Dis.

[10] Gasink LB, Edelstein PH, Lautenbach E, Synnestvedt M, Fishman NO (2009). Risk factors and clinical impact of Klebsiella pneumoniae carbapenemase-producing K. Pneumoniae. Infect Control Hosp Epidemiol.

[11] Kwak YG, Choi SH, Choo EJ, Chung JW, Jeong JY, Kim NJ (2005). Risk factors for the acquisition of carbapenem-resistant Klebsiella pneumoniae among hospitalized patients. Microb Drug Resist.

[12] Orsi GB, Bencardino A, Vena A, Carattoli A, Venditti C, Falcone M (2013). Patient risk factors for outer membrane permeability and KPC-producing carbapenem-resistant Klebsiella pneumoniae isolation: results of a double case-control study. Infection.

[13] Patel G, Huprikar S, Factor SH, Jenkins SG, Calfee DP (2008). Outcomes of carbapenem-resistant Klebsiella pneumoniae infection and the impact of antimicrobial and adjunctive therapies. Infect Control Hosp Epidemiol.

[14] Schwaber MJ, Klarfeld-Lidji S, Navon-Venezia S, Schwartz D, Leavitt A, Carmeli Y (2008). Predictors of carbapenem-resistant Klebsiella pneumoniae acquisition among hospitalized adults and effect of acquisition on mortality. Antimicrob Agents Chemother.

[15] Patel R, Paya CV (1997). Infections in solid-organ transplant recipients. Clin Microbiol Rev.

[16] Clinical and Laboratory Standards Institute. CLSI (2017). Performance standards for antimicrobial susceptibility testing.

[17] Administration, US Food and Drug. Tygacil® label information. https://www.accessdata.fda.gov/drugsatfda_docs/label/2013/021821s026s031lbl.pdf

[18] Versalovic J, Koeuth T, Lupski JR (1991). Distribution of repetitive DNA sequences ineubacteria and application to fingerprinting of bacterial genomes. Nucleic Acids Res.

[19] Ben-David D, Kordevani R, Keller N, Tal I, Marzel A, Gal-Mor O, Maor Y, Rahav G (2012). Outcome of carbapenem resistant Klebsiella pneumoniae bloodstream infections. Clin Microbiol Infect.

[20] Zarkotou O, Pournaras S, Tselioti P, Dragoumanos V, Pitiriga V, Ranellou K, Prekates A, Themeli-Digalaki K, Tsakris A (2011). Predictors of mortality in patients with bloodstream infections caused by KPC-producing Klebsiella pneumoniae and impact of appropriate antimicrobial treatment. Clin Microbiol Infect.

[21] Dellinger RP, Levy MM, Rhodes A, Annane D, Gerlach H, Opal SM (2013). Surviving Sepsis campaign guidelines committee including the pediatric subgroup. Surviving Sepsis campaign: international guidelines for management of severe sepsis and septic shock, 2012. Intensive Care Med.

[22] Hu FP, Guo Y, Zhu DM, Wang F, Jiang XF, Xu YC (2017). CHINET surveillance of bacterial resistance across China: report of the results in 2016. Chin J Infect Chemother.

[23] Hu FP, Guo Y, Zhu DM, Wang F, Jiang XF, Xu YC (2016). Resistance trends among clinical isolates in China reported from CHINET surveillance of bacterial resistance, 2005-2014. Clin Microbiol Infect.

[24] McKeage K, McCormack PL (2010). Basiliximab: a review of its use as induction therapy in renal transplantation. Bio Drugs.

[25] Van Gelder T, Hesselink DA (2015). Mycophenolate revisited. Transpl Int.

[26] Garnock-Jones KP (2015). Tacrolimus prolonged release (Envarsus®): a review of its use in kidney and liver transplant recipients. Drugs.

[27] Gupta N, Limbago BM, Patel JB, Kallen AJ (2011). Carbapenem-resistant Enterobacteriaceae: epidemiology and prevention. Clin Infect Dis.

[28] Gomez-Simmonds A, Greenman M, Sullivan SB, Tanner JP, Sowash MG, Whittier S (2015). Population structure of Klebsiella pneumoniae causing bloodstream infections at a new York City tertiary care hospital: diversification of multidrug-resistant isolates. J Clin Microbiol.

[29] Alicino C, Giacobbe DR, Orsi A, Tassinari F, Trucchi C, Sarteschi G (2015). Trends in the annual incidence of carbapenem-resistant Klebsiella pneumoniae bloodstream infections: an 8-year retrospective study in a large teaching hospital in northern Italy. BMC Infect Dis.

[30] Daikos GL, Tsaousi S, Tzouvelekis LS, Anyfantis I, Psichogiou M, Argyropoulou A (2014). Carbapenemase-producing Klebsiella pneumoniae bloodstream infections: lowering mortality by antibiotic combination schemes and the role of carbapenems. Antimicrob Agents Chemother.

[31] Lanini S, Costa AN, Puro V, Procaccio F, Grossi PA, Vespasiano F (2015). Donor-recipient infection (DRIn) collaborative study group. Incidence of carbapenem-resistant gram negatives in Italian transplant recipients: a nationwide surveillance study. PLoS One.

[32] Sousa SR, Galante NZ, Barbosa DA, Pestana JO (2010). Incidence of infectious complications and their risk factors in the first year after renaltransplantation. J Bras Nefrol.

[33] Fernández-Ruiz M, Andrés A, López-Medrano F, González E, Lumbreras C, San-Juan R, Morales JM, Aguado JM (2013). Infection risk in kidney transplantation from uncontrolled donation after circulatory death donors. Transplant Proc.

[34] Kalpoe JS, Sonnenberg E, Factor SH, del Rio MJ, Schiano T, Patel G, Huprikar S (2012). Mortality associated with carbapenem-resistant Klebsiella pneumoniae infections in liver transplant recipients. Liver Transpl.

[35] Freire MP, Abdala E, Moura ML, de Paula FJ, Spadão F, Caiaffa-Filho HH (2015). Risk factors and outcome of infections withKlebsiella pneumoniae carbapenemase-producing K. Pneumoniae in kidney transplant recipients. Infection.

[36] Xu MJ, Xie XB, Peng LK, Peng FH, Lan GB, Yu SJ (2017). Clinical analysis of 5 cases of infectious renal artery rupture after renal transplantation. Chin J Organ Transplant.

[37] Tumbarello M, Viale P, Viscoli C, Trecarichi EM, Tumietto F, Marchese A (2012). Predictors of mortality in bloodstream infections caused by Klebsiellapneumoniae carbapenemase-producing K. Pneumoniae: importance of combinationtherapy. Clin Infect Dis.

[38] Qureshi ZA, Paterson DL, Potoski BA, Kilayko MC, Sandovsky G, Sordillo E (2012). Treatment outcome of bacteremia due toKPC-producing Klebsiella pneumoniae: superiority of combination antimicrobialregimens. Antimicrob Agents Chemother.

[39] De Pascale G, Montini L, Pennisi M, Bernini V, Maviglia R, Bello G (2014). High dose tigecycline in critically ill patients with severe infections due to multidrug-resistant bacteria. Crit Care.

[40] Daikos GL, Markogiannakis A (2011). Carbapenemase-producing Klebsiella pneumoniae: (when) might we still consider treating with carbapenems?. Clin Microbiol Infect.

[41] Tian L, Tan R, Chen Y, Sun J, Liu J, Qu H (2016). Epidemiology of Klebsiella pneumoniae bloodstream infections in a teaching hospital: factors related to the carbapenem resistance and patient mortality. Antimicrob Resist Infect Control.

